# Congenitally Missing Lateral Incisors: Prioritizing Space Closure Whenever Feasible

**DOI:** 10.7759/cureus.74471

**Published:** 2024-11-26

**Authors:** Ahmed Khalil, Rawan Alrehaili, Refal Almatrodi, Abdullah Koshak, Bashayr Tawakkul, Taif Almuqati, Jihan Alharbi, Ahmed Alsaleh, Madhawi Alharbi, Saleh Al Mahfouz

**Affiliations:** 1 Dentistry, Private Sector, Medina, SAU; 2 Dentistry, Private Sector, Riyadh, SAU; 3 Dentistry, King Abdulaziz University, Jeddah, SAU; 4 Dentistry, Umm Al-Qura University, Makkah, SAU; 5 Dentistry, King Khalid University, Abha, SAU; 6 Dentistry, King Saud Bin Abdulaziz University for Health Sciences, Riyadh, SAU; 7 Orthodontics and Dentofacial Orthopedics, Private Sector, Riyadh, SAU

**Keywords:** implant, missing maxillary lateral incisor, orthodontic space closure, prosthetic replacement, temporary anchorage device

## Abstract

Congenital missing teeth are among the most prevalent dental malformations. Maxillary lateral incisors are particularly prone to agenesis, often missing bilaterally. This condition presented complex challenges for both patients and clinicians. The etiology of maxillary lateral incisor agenesis is multifactorial, involving genetic and environmental factors. The management is also inherently complex, necessitating an interdisciplinary approach. Treatment options include orthodontic space closure, resin-bonded bridges, implants, removable partial dentures, and autotransplantation. Among these, implant placement and orthodontic space closure are most commonly preferred by clinicians. The selection of an appropriate treatment modality depends on multiple factors, such as patient growth, available space, condition of adjacent teeth, and initial malocclusion. This review aimed to evaluate and compare various treatment options for maxillary lateral incisor agenesis, providing a comprehensive analysis of the latest evidence and highlighting the benefits and limitations of each approach to inform clinical decision-making.

## Introduction and background

Congenital missing teeth are one of the most common malformations, with a prevalence in permanent dentition that varies globally [1]. Maxillary lateral incisors are the most frequently affected teeth in the anterior region and are often missing bilaterally [2]. The etiology of maxillary lateral incisor agenesis is complex, involving both genetic and environmental factors, including gene mutations such as MSX and PAX9, trauma, infections, medications, and syndromes like ectodermal dysplasia, Down syndrome, and cleft lip and palate [3,4]. Patients with congenitally missing teeth may face significant esthetic challenges, which can negatively impact their self-esteem, communication, and professional life.

Management of missing maxillary anterior teeth, particularly maxillary lateral incisors, is a complex and often challenging task that requires an interdisciplinary approach. Treatment options include orthodontic space closure, resin-bonded bridges, implants, removable partial dentures, and autotransplantation [5,6]. Among these, implant placement and orthodontic space closure are the most favored by clinicians [7]. The choice of treatment depends on various factors, including the patient’s growth, amount of space available, condition of neighboring teeth, initial malocclusion, amount of gingival exposure, patient’s profile, and gingival architecture [8,9]. Implant substitution is often a common solution due to its ability to achieve good occlusion and the advantage of preserving adjacent teeth [10,11]. However, orthodontic space closure, which involves protraction of the buccal segment, also offers long-term esthetic and functional benefits [12]. One of the significant advantages of space closure is that treatment can be completed immediately after orthodontics without the need to wait for the patient’s growth to cease, which is particularly beneficial in children and adolescents [13]. Additionally, the natural result of space closure ensures that any long-term changes will occur naturally, unlike with the presence of an implant-supported crown [14]. It is also worth mentioning that dental implants, which behave similarly to ankylosed teeth by remaining in a fixed position, can lead to progressively worsening esthetic issues as the adjacent natural teeth continue to erupt [15]. This phenomenon may persist throughout life in many adult patients [16]. Furthermore, the long-term effects of the implant included a range of undesirable complications, such as peri-implantitis, peri-implant marginal bone loss, and esthetic concerns like gingival recession or discoloration [17-19]. To mitigate these complications, the use of temporary anchorage device-supported pontics was proposed as a temporary replacement solution for growing patients [20]. As treatment techniques continue to evolve, it is important to assess whether advancements have varied across different methods of treatment. Given the distinct clinical challenges associated with treating missing maxillary lateral incisors, it is also crucial to gather more comprehensive information on the management of this condition. This review aimed to examine and compare various treatment options for missing lateral incisors, evaluating them based on the latest evidence. It aims to highlight the advantages and disadvantages of each approach while offering a comprehensive overview of the current literature on this topic.

## Review

Search strategy

An extensive search of the literature was conducted using various established electronic databases, such as PubMed, Embase, MEDLINE, and Google Scholar, to identify peer-reviewed articles relevant to this review as of May 1, 2024. The search included original research articles, review papers, systematic reviews, clinical trials, and animal studies published in English. The screening process began with an initial assessment of titles and abstracts for relevance. Articles that passed this stage underwent a full-text review to verify their relevance to the review's focus. Publications not in English, studies on unrelated topics, opinion articles, and editorials with limited relevance were excluded from the search.

Prevalence of agenesis

While any tooth can be affected by agenesis, the maxillary lateral incisors, second premolars, and third molars are the most common congenitally missing teeth [21,22]. The prevalence of hypodontia in primary dentition is notably low, typically ranging from 0.1% to 0.9% of the population [23]. However, in permanent dentition, prevalence values vary widely across different populations. For instance, studies have reported prevalence rates of 7.8% in Danish and 7.9% in Icelandic populations [24,25]. A slightly lower prevalence was detected in the Turkish population at 7.5% [26]. In contrast, lower prevalence rates were reported in Sudanese, Saudi, and Qatari populations, with rates of 5.1%, 4.85%, and 6.2%, respectively [27-29]. On the higher end, two German studies reported prevalence rates of 12.6% [30] and 11.3% [31], while very low prevalence rates were noted in studies conducted in France (1.9%) and Malaysia (2.8%) [32,33]. These variations suggest significant differences between populations, likely due to factors such as geographic, racial, and ethnic origins.

Gender differences in hypodontia were examined, revealing that females exhibited a higher prevalence than males [34,35]. Additionally, most researchers found that bilateral congenitally missing teeth were more common than unilateral cases, with bilateral occurrences being nearly twice as frequent [36,37].

Orthodontic space closure with canine substitution vs. space opening with prosthodontic replacement: a puzzle yet to be resolved

When maxillary lateral incisors are congenitally missing, treatment options include orthodontically closing the space with canine substitution, prosthetic replacement, or autotransplantation. The most common approaches are either closing the space with orthodontics or creating space for a prosthetic replacement [38,39]. The prosthetic replacement includes cantilevered resin-bonded bridges, conventional prostheses, or implant-anchored prostheses [40].

Esthetic perception

The aesthetic outcomes of orthodontic space closure versus prosthetic replacement in patients with congenitally missing maxillary lateral incisors have been extensively studied. In a retrospective study, Robertsson and Mohlin [41] found that patients who underwent space closure reported higher satisfaction with their esthetic appearance compared to those who received prosthetic replacements. Additionally, space closure was associated with better periodontal health, while prosthetic solutions were linked to increased plaque accumulation and a higher incidence of gingivitis, making space closure the preferred option from both an esthetic and periodontal health perspective. Similarly, De Marchi et al. [42] evaluated smile attractiveness through assessments by both dentists and laypersons. They found that patients treated with space closure and canine substitution were more satisfied with their smiles than those who received implant-supported prostheses. Although no significant differences in smile attractiveness were observed between the two groups, there was a slight patient preference for the natural appearance achieved through orthodontic space closure. Pini et al. [43] further explored these differences, noting that patients treated with implants had lower width/length ratios for the lateral incisors compared to those who underwent space closure. Despite the space closure group showing greater discrepancies in gingival zenith positioning, which could have impacted the final esthetic appearance, both treatment methods were deemed capable of achieving satisfactory esthetic results. However, careful consideration was recommended for the specific esthetic challenges posed by each approach. A follow-up study by Pini et al. [44] reinforced these findings, demonstrating that space closure with canine substitution resulted in esthetic parameters closely resembling those of individuals without agenesis. Patients treated with space closure exhibited width/height ratios and apparent contact dimensions more in line with esthetic norms compared to those treated with implant-supported prostheses. Despite the implant group showing smaller width/height ratios and greater apparent contact dimensions, space closure proved more favorable in achieving esthetic harmony, particularly in maintaining gingival zenith positioning. In a study evaluating esthetic perceptions, both laypersons and patients consistently perceived that space closure resulted in a more favorable esthetic outcome compared to tooth-supported or implant-supported dental prostheses [45]. When substituting a missing maxillary lateral incisor with the adjacent canine, the color of the canine crown can be a significant esthetic concern, potentially impacting the overall appearance [46]. As a result, individual bleaching or laminate veneer may be considered in certain cases to improve the esthetic outcome [47].

Overall, these studies collectively suggest that orthodontic space closure may offer superior esthetic outcomes compared to prosthodontic replacement in cases of maxillary lateral incisor agenesis. This makes it the preferred treatment option for achieving esthetic harmony.

Periodontal condition and temporomandibular joint (TMJ) health

In a retrospective study by Nordquist and McNeill [48], it was found that patients who underwent orthodontic space closure experienced significantly healthier periodontal conditions compared to those who received prosthetic replacements. Patients who received fixed partial dentures were associated with higher levels of plaque accumulation and gingival inflammation. These findings were echoed in a study by Robertsson and Mohlin [41], which also reported better periodontal health outcomes with space closure, noting reduced plaque and gingivitis without compromising TMJ function. Rosa et al. [47] further supported these conclusions, demonstrating that orthodontic space closure achieved through first premolar intrusion and canine extrusion did not lead to periodontal deterioration or an increased risk of TMJ disorders when compared to a control group without missing teeth. Their findings indicated that space closure maintained periodontal health with no significant differences in probing depths, bleeding on probing, or tooth mobility between the treated and control groups. Additionally, no adverse effects on TMJ function were observed. In contrast, De Marchi et al. [49] compared periodontal and TMJ health outcomes between orthodontic space closure with tooth recontouring and implant placement. They found no significant differences in periodontal health or TMJ disorder incidence between the two treatment methods. This suggests that both approaches can maintain periodontal health and TMJ function, though earlier studies imply space closure may have slight advantages. Amm et al. [50] expanded on this by evaluating canine substitution using skeletal anchorage in patients with maxillary lateral incisor agenesis and class I or class III skeletal malocclusions. The study found that this method effectively achieved proper occlusion, improved soft tissue profiles, and maintained periodontal health and TMJ function, further supporting the efficacy of space closure techniques. Silveira et al. [51] reinforced the preference for space closure by highlighting its favorable periodontal outcomes and esthetic advantages over prosthodontic replacements. They noted lower scores in periodontal indices such as gingival inflammation and plaque accumulation in patients who underwent space closure.

Additionally, it is important to consider the complications associated with prosthodontic replacements, particularly dental implants. Gingival discoloration is a recognized complication in patients treated with implants. A study conducted by Hedmo et al. [14] reported that 60% to 70% of implant cases for missing maxillary lateral incisors resulted in discolored gingiva. In the long term, uneven gingival margins, exposure of the implant, and infraocclusion have also been reported as potential complications [52-54]. Infraocclusion of the implant was observed as a gradual discrepancy in height between the implant crown and the neighboring teeth over time, due to the continuous eruption of adjacent teeth, even in early adulthood [55,56]. This issue is particularly significant from an aesthetic perspective, especially in patients with gumminess or when a unilateral replacement is involved [57,58]. Some studies examining space closure with canine substitution did not address the use of bonded retainers, even though it is well documented that spaces may reopen and bonding is recommended to ensure long-term stability [6,59].

Overall, these studies collectively suggest that orthodontic space closure offers consistent advantages in maintaining periodontal health and TMJ function while also providing esthetically pleasing results, making it a preferred treatment option over prosthodontic replacement in many cases.

**Figure 1:**
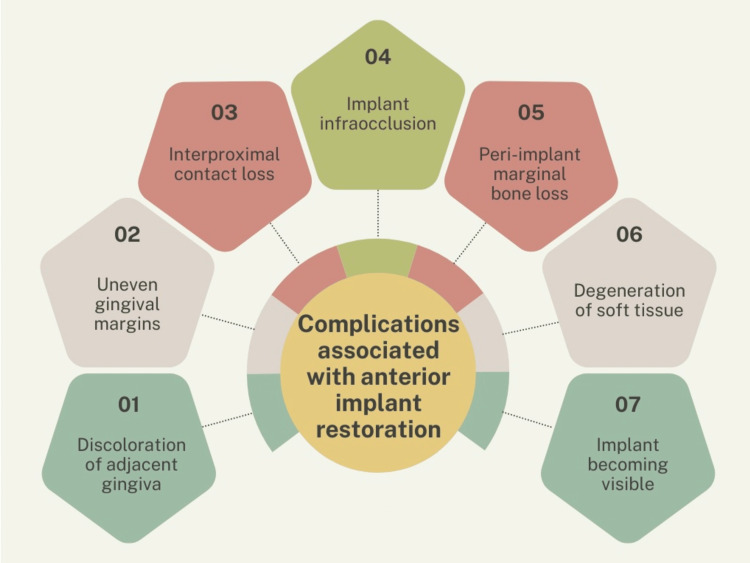
Summary of key complications in anterior implant restorations Image Credit: Authors

A summary of the complications associated with anterior implant restoration is depicted in Figure 1.

Cost analysis

In the majority of cases where a missing tooth needs to be replaced, pre-prosthetic orthodontic treatment is required to adjust and redistribute the spaces. The cost differences between canine substitution and implant placement for patients with missing maxillary lateral incisors have only been investigated in one study. Hedmo et al. [60] conducted a cost analysis comparing orthodontic space closure and implant placement for patients missing maxillary lateral incisors. The study examined both direct and indirect costs over a 12-year period, adopting a societal perspective. The findings revealed that orthodontic space closure was associated with significantly lower total societal costs compared to implant placement. While orthodontic space closure had higher initial treatment costs, it resulted in fewer long-term expenses, making it a more cost-effective option overall. This suggests that orthodontic space closure may be a more economical approach for managing missing maxillary lateral incisors.

Cantilevered resin-bonded fixed prosthesis

A recent systematic review and meta-analysis by Al-Bermani et al. [61] assessed the effectiveness of using anterior single-retainer zirconia resin-bonded fixed prostheses for replacing missing anterior teeth. The findings demonstrated that these prostheses offer a dependable and long-lasting solution when proper bonding techniques are employed. The study reported high success rates over extended follow-up periods with minimal complications. These results support the use of single-retainer zirconia prostheses as a favorable treatment option for anterior tooth replacement, combining both functional and esthetic benefits. Similarly, Kern et al. [62] concluded that all-ceramic cantilever resin-bonded fixed dental prostheses offer an excellent minimally invasive alternative to implants and traditional prosthetic techniques for the replacement of single missing anterior teeth. Spinas et al. [63] further supported this by highlighting that all-ceramic cantilever resin-bonded fixed dental prostheses are particularly well-suited for adolescents or young adults who may still be experiencing growth. Tanoue [64] highlighted the importance of a patient's age at the time of insertion of resin-bonded fixed dental prostheses, noting that the risk of failure in younger patients was 1.7 times greater than that in older patients. This increased risk was primarily associated with a higher likelihood of trauma in younger individuals. Conversely, King et al. [65] reported that patients under 30 years of age actually exhibited a lower failure rate. Moreover, they also found a significantly higher success rate for those who were placed using a rubber dam. Zirconia's flexural strength, which is double that of glass-infiltrated alumina ceramic, allows for the possibility of rebonding zirconia resin-bonded fixed dental prostheses without complications [66]. In contrast, alumina resin-bonded fixed dental prostheses that experience failures or fractures in the framework typically necessitate a complete replacement restoration [62]. While some studies advocated for a "no preparation" approach [67,68], the majority recommended creating grooves, pits, slots, chamfers, and proximal boxes on the lingual/palatal surfaces of the abutment teeth to ensure proper seating and retention of the prostheses [69-71].

An overview of studies evaluating the survival rates of resin-bonded fixed dental prostheses (RBFDPs) is presented in Table 1.

**Table 1:** Overview of studies assessing the survival rates of resin-bonded fixed dental prostheses (RBFDPs)

Study author and year	Study design	Type and material of prostheses	Follow-up time	Survival rate
Sun et al. [72] 2013	Case series	Cantilevered IPS e.max press veneer-retained fixed partial denture	3.8 years	100%
Saker et al. [73] 2014	Retrospective study	Anterior metal-ceramic and all-ceramic cantilever resin-bonded fixed prostheses	5 years	100% (metal-ceramic); 90% (all-ceramic)
Galiatsatos and Bergou [74] 2014	Prospective clinical study	All-ceramic resin-bonded fixed dental prostheses (glass-infiltrated alumina)	8 years	85.18%
Kumbuloglu and Ozcan [75] 2015	Prospective clinical study	Indirect, anterior 3-unit surface-retained fiber-reinforced-composite prostheses	7.5 years	97.7%
Naenni et al. [76] 2020	Retrospective study	Zirconia ceramic single retainers, resin-bonded fixed dental prostheses	10 years	100%

Restorative considerations and finishing

When the treatment plan involves closing maxillary spaces, it is crucial to include a restorative dentistry specialist in the process. This collaboration is essential for achieving the best aesthetic and functional outcomes, as restorative esthetic finishing often necessitates the reshaping of canines. For an optimal result, the torque of the reshaped canines should closely resemble that of the lateral incisors. Additionally, palatal movement of the canine root can reduce alveolar bone prominence, enhance interproximal contact with the central incisor, and decrease the occlusal load on mandibular incisors [77]. Toward the end of space closure treatment, particular attention should be given to the maxillary first premolar. Slight mesial rotation and intrusion of the crown are recommended to eliminate interferences on both the working and balancing sides, reduce the need for cusp wear, and conceal the palatal cusp. Moreover, it is essential to anticipate and address potential tooth-size discrepancies during the planning phase, as these discrepancies can significantly affect the quality of intercuspation [78,79]. In cases where maxillary permanent lateral incisors are missing and the deciduous incisors are retained, the study by Giachetti et al. [80] introduced the biologically active intrasulcular restoration technique as an innovative and conservative solution. This approach allowed for the transformation of the deciduous lateral incisor into a permanent-like tooth by modifying its morphology, proportions, and the appearance of the surrounding soft tissues. It offered a viable, non-invasive alternative for rehabilitating such cases, effectively managing the esthetic and functional challenges associated with congenital maxillary lateral incisor agenesis.

**Figure 2:**
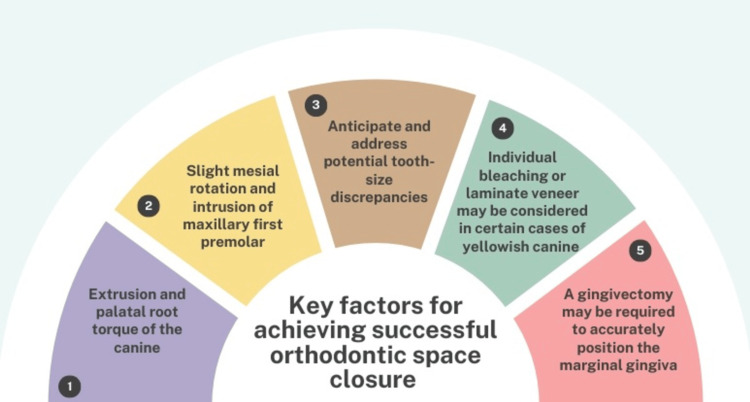
Summary of the critical elements needed for effective orthodontic space closure Image Credit: Authors

An overview of the key factors for achieving successful orthodontic space closure is depicted in Figure 2.

Use of temporary anchorage device-supported pontics in growing patients

Given that osseointegrated dental implants are unsuitable for patients who are still growing [20,81], provisional restorations are necessary for children and adolescents with missing permanent maxillary lateral incisors following orthodontic treatment with space opening until they have completed their growth. Removable appliances with pontics are often not well-received by patients and tend to deliver less satisfactory esthetic results [82]. Moreover, the lack of alveolar ridge loading can lead to alveolar bone atrophy, which may necessitate periodontal procedures before implant placement [83-85]. An alternative solution proposed for temporarily replacing missing permanent maxillary lateral incisors in growing patients is the use of mini-screw-supported pontics [86]. This approach offers several potential benefits, such as preserving alveolar bone height and width, maintaining the quality and quantity of alveolar bone (Melsen et al. 2015), preventing ridge atrophy (Ostler and Kokich 1994), and ensuring space maintenance (Cope and McFadden 2014; Kalia 2015; Graham 2007) [87,88]. Additionally, this method does not require the preparation of neighboring teeth, allows for easy placement and removal of the temporary anchorage device, and facilitates immediate dental implant placement after temporary anchorage device removal once the patient's growth ceases [89].

Limitations

The study faced several challenges, including inconsistencies between the comparison groups, the absence of a calculated sample size, the lack of blinding in the evaluation process where it could have been applied, and relatively short periods for post-treatment assessments. It is noteworthy that much of the available evidence on the use of temporary anchorage device-supported pontics for the transitional management of missing permanent maxillary lateral incisors in children and adolescents came from case reports or series, which often did not follow a standardized surgical and prosthetic protocol for temporary anchorage device placement and loading [90,91].

## Conclusions

Implants and tooth-supported dental prostheses for maxillary lateral incisor agenesis tend to have poorer outcomes in both periodontal health indicators and esthetic evaluations compared to orthodontic space closure. Hence, it can be recommended that orthodontic space closure should be the preferred option, provided the diagnostic characteristics are favorable. The use of pontics supported by temporary anchorage devices has been proposed as a provisional replacement for growing patients. The findings of the present review should be considered alongside the expectations of the patients, the expertise of the professional team, as well as any financial limitations, in order to determine the most appropriate treatment for each case. The selection of the treatment modality should focus on the patient, be grounded in evidence, and strive to minimize the lifelong treatment burden.
